# Preoperative Fibrosis-4 (FIB-4) Evaluation May Be Helpful to Evaluate Prognosis of Gastric Cancer Patients Undergoing Operation: A Retrospective Study

**DOI:** 10.3389/fonc.2021.655343

**Published:** 2021-06-17

**Authors:** Ke Xu, Mingming Shi, Weiteng Zhang, Yiyi Shi, Qiantong Dong, Xian Shen, Xiaolei Chen, Ji Lin

**Affiliations:** ^1^ IT Department, The First Affiliated Hospital, Wenzhou Medical University, Wenzhou, China; ^2^ Department of Gastrointestinal Surgery, The First Affiliated Hospital, Wenzhou Medical University, Wenzhou, China; ^3^ Department of Anesthesiology, The First Affiliated Hospital, Wenzhou Medical University, Wenzhou, China; ^4^ Department of General Surgery, The Second Affiliated Hospital and Yuying Children’s Hospital of Wenzhou Medical University, Wenzhou, China

**Keywords:** gastric cancer, FIB-4 index, liver function, overall survival, platelet

## Abstract

**Background:**

Liver dysfunction and chronic inflammation influence the prognosis of many tumors and surgical outcomes. This study was performed to determine whether the Fibrosis-4 (FIB-4) index, originally defined as a noninvasive fibrosis marker, can predict the prognosis of patients with gastric cancer undergoing radical gastric cancer surgery.

**Methods:**

We have retrospectively analyzed 594 consecutive patients with gastric cancer who underwent gastrectomy in our database. The FIB-4 index was calculated using laboratory data and age before gastrectomy. The clinical utility of FIB-4 was evaluated by X-tile. Patients were divided into two groups (high and low FIB-4 index groups), and their overall survival (OS) was investigated. Cox regression analysis was used to identify the independent parameters associated with prognosis. Finally, we developed a prognostic prediction model by using R statistical software.

**Results:**

A total of 556 patients, including 422 men and 134 women, were enrolled. Of these, 61 (11.0%) and 495 (89.0%) patients had low and FIB-4 indexes, respectively. In addition to the indicators of FIB-4, preoperative age, tumor site, surgical procedure, TNM stage, and postoperative complications were found to be independent predictors of prognosis (*P* < 0.05). Among patients, the FIB-4 index group had significantly shorter OS (log-rank *P* = 0.01) than the low FIB-4 index group. This association was also confirmed in the multivariate analysis (hazard ratio, 4.65; 95% confidence interval, 1.07-4.29; *P* = 0.031).

**Conclusions:**

Preoperative FIB-4 index can predict long-term outcomes of gastric cancer patients who had undergone gastrectomy.

## Introduction

Currently, gastric cancer is the fifth most common cancer and the third most common cause of cancer death globally with over 1 million estimated new cases; and nearly 800,000 people die of this disease annually (1). Surgery is still currently considered to be the only radical treatment. As surgical techniques improve and progress is made in traditional radiotherapy, chemotherapy, and implementation of neoadjuvant therapy, the 5-year survival rate of patients with early gastric cancer can reach >95% (2). However, because patients are mostly asymptomatic in the early stages of the disease, most of them have an advanced-stage disease at diagnosis; thus, the best timing for surgery is missed, and the prognosis of gastric cancer patients remains poor, especially east Asians (1, 3). At present, there is insufficient preoperative intervention and treatment for patients with poor prognosis. Pathological TNM stage is recognized as the best prognostic model, but it still has shortcomings.

The morbidity of liver cirrhosis is probably higher than that reported, due to the compensatory function of the liver; thus, patients at an early stage of cirrhosis are frequently asymptomatic and often undiagnosed (4). Likewise, liver-related mortality is allegedly underestimated, partly because the determination of liver-related death is incomplete. These defects indicate that the burden of chronic liver disease should include deaths due to hepatobiliary cancers and hepatitis for the accurate determination of liver-related deaths (5–9). The relative mortality of decompensated liver cirrhosis was even greater than that of gastric cancer (10).

The Fibrosis-4 (FIB-4) index, calculated as age × aspartate aminotransferase/(platelet count × √alanine aminotransferase), was developed as a noninvasive index to stage hepatic disease in patients with viral infection (11), and it is a simple and inexpensive measure of hepatic disorder. This index has been used across many hepatic diseases (12–14), and several studies have described the association between a high FIB4 index and poorer outcomes (15, 16), not only for hepatic disease but also for non-hepatic disease. Previous studies reported that the FIB4 index was associated with long-term mortality and readmission rate of heart failure patients (17, 18). Some reports have shown that the FIB-4 index is not only a predictor of background liver fibrosis but also a prognostic factor after hepatectomy in patients with colorectal cancer liver metastases (19). However, there is no evidence that the FIB-4 index can predict the long-term outcomes of patients with operable gastric cancer. Thus, the aim of the present study was to evaluate the predictive value of the FIB-4 index at admission for adverse outcomes in patients with operable gastric cancer.

## Methods

### Study Population

We retrospectively identified patients diagnosed with operable gastric cancer between January 1, 2014 and December 31, 2016 at the First Affiliated Hospital of Wenzhou Medical University, Zhejiang, China. All patients had histologically confirmed gastric cancer. The inclusion criteria were as follows: (1) had undergone radical gastrectomy and (2) had undergone blood examinations <2 weeks prior to the operation. The exclusion criteria included (a) occurrence of another malignancy during the 3 years prior to surgery; (b) had undergone an emergent operation; and (c) had received preoperative chemotherapy or radiotherapy. After applying the abovementioned criteria, 35 patients were excluded. Moreover, four patients who died within 30 days after surgery, 19 patients who were lost to follow-up, ten patients affected the outcome of death because of other causes, and five patients with missing preoperative aspartate aminotransferase (AST), alanine aminotransferase (ALT), or platelet (PLT) data were also excluded. Finally, 556 patients were included in the final analysis.

### Perioperative Factors

The following data were collected and recorded: patients’ personal information (i.e., age, sex, body mass index [BMI]), blood examination data (routine blood parameters, biochemical indexes), and tumor characteristics (i.e., location, histopathological differentiation). The diagnoses were confirmed in all patients by histological examination. The American Society of Anesthesiologists (ASA) grade (according to the standard proposed by the ASA), surgical history, and other factors were collected prior to surgery. Nutritional risk screening (NRS) 2002 was utilized for preoperative nutritional risk assessment within 24 h of admission (20). The type of surgical resection, extent of lymph node dissection, and determination of disease stage were selected according to the Japanese Gastric Cancer Association (14th edition) (21).

### FIB-4 Evaluation

Blood specimens were obtained within 14 days prior to surgery and translocated to sterile centrifuge tubes, which were carefully delivered to the clinical laboratory department. A hemocounter (XE2100; Sysmex Co., Kobe, Japan) was used to calculate the platelet. Blood biochemical items including AST and ALT were also calculated. The FIB-4 index was calculated using the following formula:

$$\begin{array}{l} \text{age}\;(\text{yrs})\times \text{aspartate}\;\text{aminotransferase}\;(\text{AST})\;[\text{U}/\text{L}]/(\text{platelets}\;\text{count}\;[10\text{ˆ}9/µ\text{L}]\\ \times \;\surd \text{alanine}\;\text{aminotransferase}\;(\text{ALT})[\text{U}/\text{L}] \end{array}$$

By using the enumeration method in X-tile (version 3.6.1; Robert L. Camp, M.D., PH.D. Yale University), the value with the maximal Youden index was chosen as the cut-off point of the preoperative FIB-4. Thus, the patients were divided into following two groups based on the cut-off point of the preoperative FIB-4: high and low FIB-4 index groups.

### Statistical Analysis

The mean and standard deviation were used for the normal distributed data, whereas the median and interquartile range were used for the non-normal distributed data. The t test was used to compare the continuous variables, such as patients’ background status, expressed as mean and standard deviation between the high and low FIB-4 index groups. The relationships between the clinicopathologic characteristics and FIB-4 were analyzed using the χ2-test or Fisher’s exact test. The OS curves in the high and low FIB-4 index groups were analyzed using the Kaplan–Meier method and compared using the log-rank test. Cox proportional hazard models were used to estimate the value of preoperative FIB-4, preoperative age, tumor site, surgical procedure, type of reconstruction, TNM, stage and postoperative complications as independent predictive indicators of prognosis. A *P* value<0.05 was considered statistically significant. Statistical analyses were performed using SPSS software version 22.0 (SPSS Inc., Chicago, IL, USA).

### Development of the Prognostic Prediction Model

We developed a prognostic prediction model, visualized it with a nomogram chart, calculated the c-index, and performed a decision curve analysis. These steps are all implemented by using R statistical software (The R Foundation, Vienna, Austria).

## Results

### Patient Characteristics

Of the 556 patients selected, 422 were men and 134 were women. The median age of the patients was 64.34 ± 10.72 years. The median FIB-4 index of the patients was 1.65 ± 0.93. According to the NRS score, 274 patients had a score of 3 or higher (38.3%). There were 460 (82.7%) patients with ASA grade I or II. Overall, 351 (63.1%) patients had tumors located in the gastric pylorus and had advanced disease (T3-4). **Table 1** reports the demographics and clinical characteristics of the patients. The prognosis was significantly poorer in the high FIB-4 index group than in the low FIB-4 index group (*P* = 0.005).

**Table 1:** Preoperative backgrounds and comparison of backgrounds based on FIB-4 index.

Variables	Overall Mean ± SD (%) [n = 556]	High FIB-4 index Mean ± SD (%) [n = 495]	Low FIB-4 index Mean ± SD (%) [n = 61]	p-Value
Age, y	64.34 ± 10.72	65.86 ± 9.46	52 ± 12.35	<0.001
Gender				0.17
Male	422 (75.9)	380(76.8)	42(68.9)	
Female	134 (24.1)	115(23.2)	19(31.1)	
BMI, kg/m^2^	22.43 ± 3.01	22.43 ± 3.01	22.49 ± 3.06	0.78
HB (g/L)	121.23 ± 22.05	122.41 ± 21.04	111.64 ± 27.33	0.003
ALB (g/L)	38.19 ± 4.42	38.14 ± 4.39	38.63 ± 4.66	0.44
PLT (10^9/L)	245.62 ± 82.40	233.32 ± 70.70	345.49 ± 101.33	0.003
ALT	18.96 ± 14.73	18.37 ± 12.83	23.77 ± 25.00	<0.001
AST	22.78 ± 10.02	23.23 ± 9.99	19.18 ± 9.60	0.59
FIB-4	1.65 ± 0.93	1.77 ± 0.91	0.65 ± 0.12	<0.001
ASA				<0.001
1-2	460(82.7)	403(81.4)	57(93.44)	
≥3	96(17.3)	92(18.6)	4(6.56)	
Charlson score				0.07
0	282(50.7)	243(49.1)	39(63.9)	
1-2	252(45.3)	231(46.7)	21(34.4)	
3-6	22(4.0)	21(4.2)	1(1.6)	
NRS				0.10
1-2	343(61.7)	302(61.0)	41(67.2)	
3-4	172(30.9)	153(30.9)	19(31.1)	
5-6	41(7.4)	40(8.1)	1(1.6)	
Surgical history				0.50
No	443(79.7)	392(79.2)	51(83.6)	
Yes	113(20.3)	103(20.8)	10(16.4)	
Abdominal surgery history				0.31
No	484(87.1)	428(86.5)	56(91.8)	
Yes	72(12.9)	67(13.5)	5(8.2)	
Preoperative diabetes				0.83
No	492(88.5)	437(88.3)	55(90.2)	
Yes	64(11.5)	58(11.7)	6(9.8)	
Hypertension				0.09
No	413(74.3)	362(73.1)	51(83.6)	
Yes	143(25.7)	133(26.9)	10(16.4)	
Hepatic diseases				1
No	534(96.0)	475(96.0)	59(96.7)	
Yes	22(4.0)	20(4.0)	2(3.3)	
Laparoscopic surgery				1
No	410(73.7)	365(73.7)	45(73.8)	
Yes	146(26.3)	130(26.3)	16(26.2)	
Surgical procedure				0.05
SG	345(62.1)	300(60.6)	45(73.8)	
TG	211(37.9)	195(39.4)	16(26.2)	
Type of reconstruction				0.10
B-I	220(39.6)	195(39.4)	25(41.0)	
B-II	91(16.4)	77(15.6)	14(23.0)	
Roux-en-Y	245(44.1)	223(45.1)	22(36.1)	
Combined resection				0.64
No	507(91.2)	450(90.9)	57(93.4)	
Yes	49(8.8)	45(9.1)	4(6.6)	
Surgical durations (min)	203.92 ± 53.61	204.13 ± 53.65		0.94
Histologic type				0.44
Undifferentiated	148(26.6)	129(26.1)	19(31.1)	
Differentiated	408(73.4)	366(73.9)	42(68.9)	
Tumor site				0.62
Upper	73(13.1)	64(12.9)	9(14.8)	
Middle	111(20.0)	101(20.4)	10(16.4)	
Low	351(63.1)	310(62.6)	41(67.2)	
Mixed	21(3.8)	20(4.0)	1(1.6)	
T stage				0.04
1-2	205(36.9)	190(38.4)	15(24.6)	
3-4	351(63.1)	305(61.6)	46(75.4)	
N stage				0.29
0	249(44.8)	221(44.6)	28(45.9)	
1	99(17.8)	93(18.8)	6(9.8)	
2	111(20.0)	97(19.6)	14(23.0)	
3	97(17.4)	84(17.0)	13(21.3)	
TNM stage				0.34
I	179(32.2)	164(33.1)	15(24.6)	
II	119(21.4)	103(20.8)	16(26.2)	
III	258(46.4)	228(46.1)	30(49.2)	
Postoperative complications				0.25
NO	370(66.5)	325(65.7)	45(73.8)	
YES	186(33.5)	170(34.3)	16(26.2)	

SD, standard deviation; BMI, body mass index; HB, hemoglobin; ALB, albumin; PLT, platelet; ALT, alanine aminotransferase; AST, aspartate aminotransferase; FIB-4, Fibrosis-4; ASA, American Society of anesthesiologists; NRS, Nutritional Risk Screening; SG, subtotal gastrectomy; TG, total gastrectomy.

### Clinicopathologic Characteristics of Gastric Cancer Associated With Preoperative FIB-4

According to the result of the analysis using the enumeration method in X-tile, the cut-off value of the preoperative FIB-4 was 0.8. On the basis of the cut-off value, the sensitivity of FIB-4 was 95%. Thus, we dichotomized the patients into the high FIB-4 (>0.8) and low FIB-4 (≤0.8) index groups. Of the 556 patients, the number of patients with a high FIB-4 index was 495 (89%). Clinicopathologic features of gastric cancer associated with preoperative FIB-4 was then further analyzed. With respect to the other clinicopathologic characteristics examined, FIB-4 was significantly associated with age (*P* < 0.001), hemoglobin (HB) (*P* = 0.003), PLT (*P* = 0.003), ALT (*P* < 0.001), ASA (*P* < 0.001), surgical procedure (*P* = 0.05), and T stage (*P* = 0.04). No significant association in the other clinicopathological characteristics was observed in our study.

### Univariate and Multivariate Analyses of Clinicopathological Characteristics and Predictive Value of the Scoring System

Univariate analysis of clinicopathological characteristics indicated that age (*P* < 0.01), FIB-4 (*P* =0.01), ASA (*P* = 0.03), NRS (*P* < 0.01), Charlson score (*P* < 0.01), albumin (ALB) (*P* < 0.01), tumor site (*P* < 0.01), laparoscopic surgery (*P* < 0.01), surgical procedure (*P* < 0.01), combined resection (*P* < 0.01), type of reconstruction (*P* < 0.01), T stage (*P* < 0.01), N stage (*P* < 0.01), TNM stage (*P* < 0.01), and postoperative complications (*P* < 0.01) showed significant differences according to prognosis (**Table 2**). There was no significant relationship found between prognosis and sex (*P* = 0.06), BMI (*P* = 0.09), previous surgery (*P* = 0.20), history of abdominal surgery (*P* = 0.23), HB (*P* = 0.09), hepatic diseases (*P* = 0.42), surgical durations (*P* = 0.74), or histologic type (*P* = 0.67).

**Table 2:** Prognostic factors for overall survival.

Variables	Univariate analysis		Multivariate analysis	
	HR (95% CI)	p value	HR (95% CI)	p value
FIB-4 (low/high)	2.54(1.22-5.28)	0.01*	4.65(1.07-4.29)	0.031*
Gender	1.54(0.98-2.43)	0.06		
Age (≧70/<70)	2.44(1.67-3.57)	<0.01*	4.66(1.03-2.00)	0.031*
BMI (≧25/<25)	0.62(0.37-1.05)	0.09		
ASA		0.03*		
NRS		<0.01*		
Charlson score		<0.01*		
Previous surgery	1.33(0.86-2.08)	0.20		
Previous abdominal surgery	1.38(0.81-2.32)	0.23		
HB (≧100g/L/<100 g/L)	1.48(0.94-2.32)	0.09		
ALB(≧35/<35 g/L)	0.43(0.28-0.64)	<0.01*		
Hepatic diseases	1.44(0.59-3.49)	0.42		
Tumor site		<0.01*	14.96	<0.01*
Upper			1	
Middle			0.01(0.59-1.82)	0.913
Low			2.09(0.87-2.54)	0.148
Mixed			11.02(1.59-5.99)	<0.01*
Laparoscopic surgery	0.37(0.23-0.61)	<0.01*		
Surgical procedure (SG/TG)	3.15(2.15-4.61)	<0.01*	5.59(1.18-5.94)	0.018*
Combined resection	3.15(1.74-5.70)	<0.01*		
Type of reconstruction		<0.01*	5.89	0.053
Roux-en-Y			1	
B-I			0.01(0.45-2.36)	0.934
B-II			2.19(0.81-4.38)	0.139
Surgical durations	1.06(0.74-1.54)	0.74		
T stage	7.06(4.16-11.98)	<0.01*		
N stage		<0.01*		
TNM stage		<0.01*	52.77	<0.01*
I			1	
II			8.72(1.46-6.53)	<0.01*
III			40.04(4.37-16.40)	<0.01*
Histologic type	0.89(0.58-1.35)	0.67		
Postoperative complications		<0.01*	5.24(1.06-2.04)	0.022*

HR, hazard ratio; CI, confidence interval; FIB-4, Fibrosis-4; BMI, body mass index; ASA, American Society of anesthesiologists; NRS, Nutritional Risk Screening; HB, hemoglobin; ALB, albumin; SG, subtotal gastrectomy; TG, total gastrectomy.

*Statistically significant (P < 0.05).

**Figure 1:**
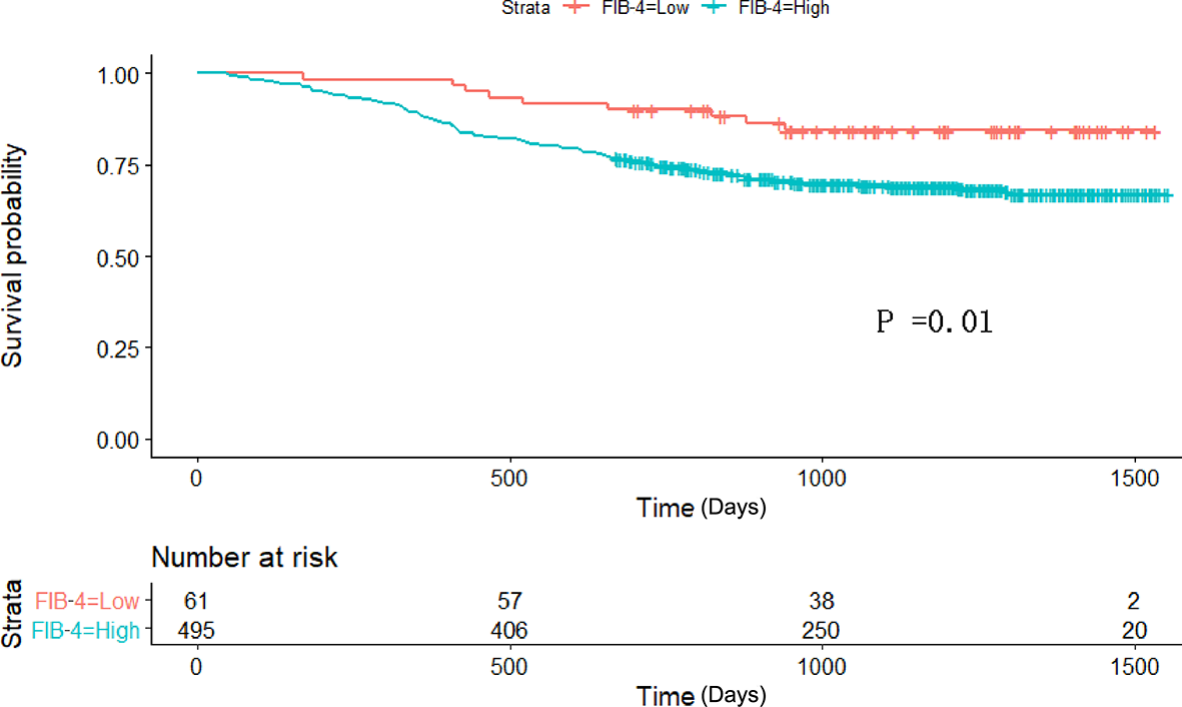
Kaplan–Meier survival curve analyses for overall survival among the 556 patients who underwent radical gastrectomy. FIB-4 Fibrosis-4.

**Figure 2:**
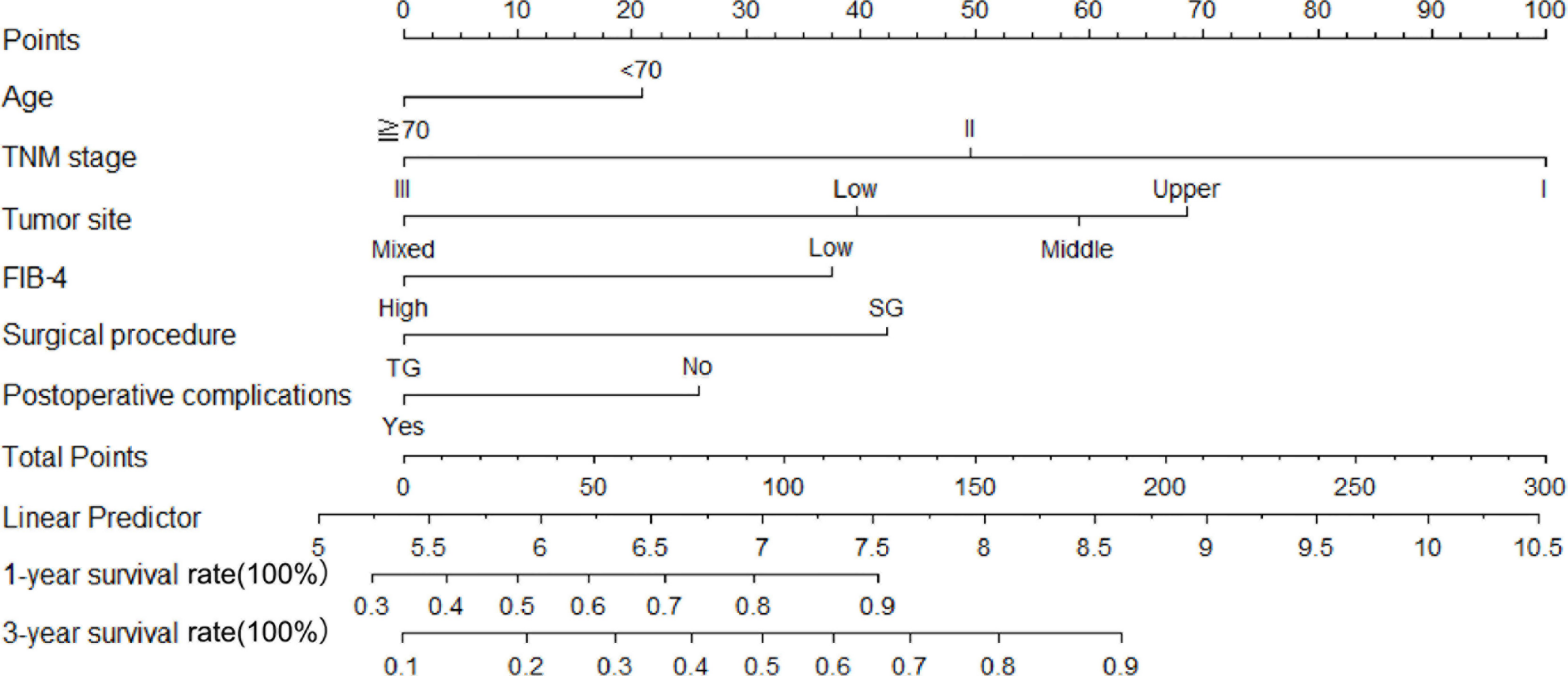
A nomogram indicating the survival. An example of a nomogram—Draw an upward vertical line from the covariate to the points bar to calculate points. Based on the sum of the covariate points, draw a downward vertical line from the total points line to calculate survival rate.

**Figure 3:**
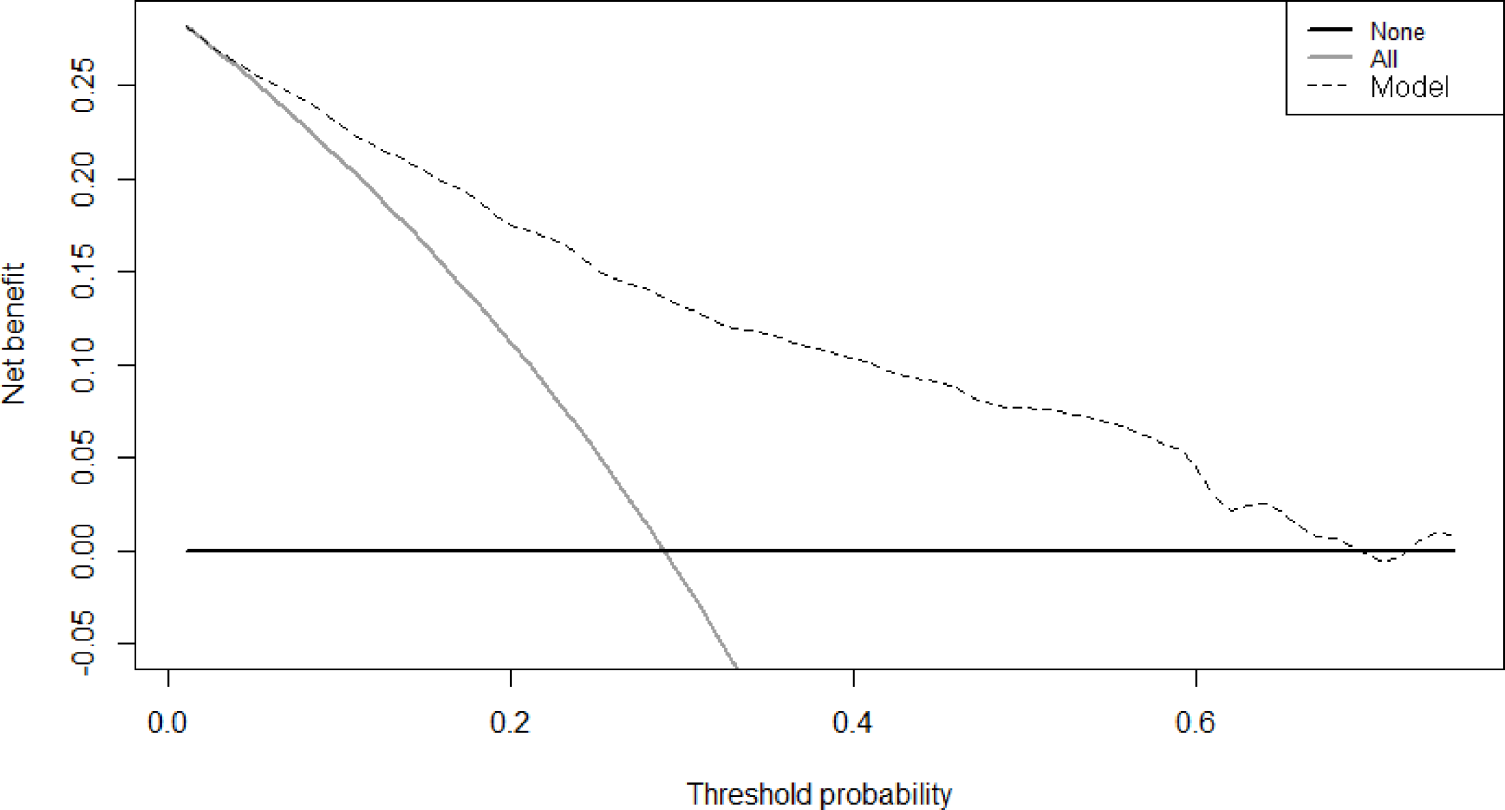
Decision curve analysis for prediction model with FIB-4.

Therefore, among the 15 variables examined in the univariate analysis (*P* < 0.05) were selected as potential independent risk factors in the multivariate analysis. The results showed (**Table 2**) that six of the 15 variables were independent predictive indicators of prognosis (*P* < 0.05), which were as follows: FIB-4 (hazard ratio [HR] 4.65; 95% confidence interval [CI] 1.07-4.29; *P* = 0.031), age (HR 4.66; 95% CI 1.03-2.00; *P* = 0.031), tumor site [(middle vs. upper: HR 0.01, 95% CI 0.59-1.82; *P* = 0.913); (low vs. upper: HR 2.09; 95%CI 0.87-2.54; *P* =0.148); (mixed vs. upper: HR 11.02; 95%CI 1.59-5.99; *P* < 0.001)], surgical procedure (HR 5.59; 95% CI 1.18-5.94; *P* = 0.018), TNM stage [(II vs. I: HR 8.72, 95% CI 1.46-6.53; *P* < 0.001); (III vs. I: HR 40.04; 95%CI 4.37-16.40; *P* < 0.001)] and postoperative complications (HR 5.24; 95% CI 1.06-2.04; *P* = 0.022) were. The OS curves between the high and low FIB-4 index groups are shown in **Figure 1** (log-rank *P* = 0.01). Finally, the model was established, c-index of the model was 0.783. (**Figure 2**) The decision curve analysis also shows the better net benefit of the model (**Figure 3**). The model has certain clinical utility.

## Discussion

This is the first study to retrospectively reveal the relationship between FIB-4 index and prognosis of patients with gastric cancer. In 556 patients with operable gastric cancer, we found that a higher FIB-4 index is associated with worse OS. Furthermore, there were direct correlations observed between prognosis and age, tumor site, surgical procedure, type of reconstruction, TNM stage, and postoperative complications. We constructed a prognostic prediction model, which can be used to predict the prognosis of gastric cancer patients after surgery and which may be useful for the timely implementation of therapeutic interventions to improve the prognosis of patients.

In this study, dichotomous and triclassification tests in X-tile are used to determine the cut-off value. Both methods showed a cutoff value of 0.8. Meanwhile, a cut-off value of 2.4 obtained by performing the triclassification test had a sensitivity of 15.7% and a specificity of 89.5%. Since FIB-4 is a screening indicator rather than a specific indicator, a cut-off value with a higher sensitivity is selected.

The FIB-4 index was used to diagnose liver cirrhosis when it was first proposed (11); its non-invasive advantages are more superior to liver puncture. In recent years, there have been more studies in the field of liver disease (12, 14, 22). “Hepatitis, cirrhosis, then liver cancer” is a trilogy of common liver diseases. It is well known that China has a large population of patients with hepatitis B (23). Along with economic development, the number of patients with fatty liver and alcoholic liver has also increased (9, 24). Perhaps there is a slight correlation between preoperative gastric cancer and moderate-to-severe cirrhosis, because of the strong compensatory ability of the liver; many gastric cancer patients with occult liver disease are asymptomatic and undiagnosed. We considered that liver fibrosis associated with cancer-associated chronic inflammation may lead to the deterioration of the systemic nutritional status and anemia-associated chronic inflammation.

FIB-4 and age are both found to be independent prognostic risk factors in this study, indicating that FIB-4 covers a part of age-independent prognostic effects. This aspect is partly manifested by liver function indicators, and the influence of platelet cannot be excluded. Platelet count is a hematological index related to the procoagulant activity of the blood. At the same time, many studies have also suggested that it is an indicator of inflammation. Platelets contribute to thromboinflammatory processes owing their capacity to interact functionally with the activated endothelium, leukocytes, and coagulation proteins; the mechanisms are multivariate (25). The factors such as TNF-α and TNF-γ released by the tumor may also cause chronic systemic inflammation and microthrombosis, which may cause abnormalities in platelet function and number (26). Predictably, because of adverse events, preoperative chemotherapy may affect the patient’s liver function; thus, the relationship between FIB-4 and prognosis of patients undergoing neoadjuvant chemotherapy before surgery should be examined further, especially that, in recent years, neoadjuvant chemotherapy has been increasingly used.

According to our research, TNM stage definitely has a considerable impact on prognosis. A mixed type of gastric cancer in the tumor site, commonly known as leather stomach or diffuse (or infiltrating) stomach cancer, indicates that the disease has locally advanced; this is a sign of poor prognosis, and histopathology often suggests poorly differentiated adenocarcinoma or even signet ring cell carcinoma (27). Compared with partial gastrectomy, total gastrectomy is performed on patients with tumors located in the upper middle of the stomach or those with a wide range of tumor locations, with Roux-en-Y being the most common anastomotic procedure performed. Total gastrectomy results in worse nutritional intake after surgery, and a previous study has suggested that the prognosis is worse in patients who had undergone total gastrectomy compared to those who had undergone subtotal gastrectomy (28).

This study has several limitations. First, this is a single-center study; thus, our findings need to be further clarified in studies involving a large sample from multiple centers. Second, the current research is a three-year outcome study; thus, the median survival time was not met and many patients dropped out at approximately 1000 days. The prognostic relevance is expected to be 5-10 years. Third, at present, neoadjuvant chemotherapy for resectable advanced gastric cancer and conversion therapy for unresectable gastric cancer are increasingly used, despite the fact that chemotherapy has a greater impact on liver function. We have not included patients who had undergone preoperative chemotherapy; thus, these patients should be included in future studies. Finally, our study had a retrospective design. There is a lack of preoperative cirrhosis data, and the relationship between FIB-4 and cirrhosis and prognosis remains unclear; additionally, no stratified analysis of liver diseases was carried out.

In conclusion, the FIB-4 index, a noninvasive liver fibrosis marker, can be an indicator of prognosis after radical gastrectomy in patients with gastric cancer. There may be a possibility of improving the prognosis of these patients through research of effective treatments to improve liver function and inflammation before surgery.

## Data Availability Statement

The original contributions presented in the study are included in the article/supplementary material. Further inquiries can be directed to the corresponding authors.

## Ethics Statement

Written informed consent was obtained from the individual(s) for the publication of any potentially identifiable images or data included in this article.

## Author Contributions

KX: acquired, analyzed, and interpreted the data, and drafted the manuscript. MS: acquired and analyzed data, and carried out the statistical analysis. YS: acquired data. WZ: carried out the statistical analysis and revised the manuscript. QD: funded the study. XS: supervised the study. XC: designed the study, analyzed and interpreted the data, supervised the study, revised the manuscript, and finally approved the version of the manuscript for publication. JL: contributed toward the conception of the study, designed the study, analyzed and interpreted the data, revised the manuscript, and finally approved the version of the manuscript for publication. All authors contributed to the article and approved the submitted version.

## Funding

This project was sponsored by the Zhejiang Provincial Health Department Medical Support Discipline- Nutrition (11-ZC), the Wenzhou Municipal Science and Bureau (Y2020732).

## Conflict of Interest

The authors declare that the research was conducted in the absence of any commercial or financial relationships that could be construed as a potential conflict of interest.
